# Confirmatory factor analysis and exploratory structural equation modeling of the factor structure of the Questionnaire of Cognitive and Affective Empathy (QCAE)

**DOI:** 10.1371/journal.pone.0261914

**Published:** 2022-02-07

**Authors:** Rapson Gomez, Taylor Brown, Shaun Watson, Vasileios Stavropoulos

**Affiliations:** 1 Department of Psychology, School of Science, Psychology and Sport, Federation University, Ballarat, Victoria, Australia; 2 Department of Psychology, College of Health and Biomedicine, Victoria University, Footscray, Victoria, Australia; University of Bologna, ITALY

## Abstract

The Questionnaire of Cognitive and Affective Empathy (QCAE) is a multiple dimensional measure of cognitive empathy [comprising primary factors for perspective taking (PT), online simulation (OS)], and affective empathy [comprising primary factors for emotion contagion (EC), proximal responsivity (PRO), and peripheral responsivity (PER)]. This study used independent clusters confirmatory factor analysis (ICM-CFA) and exploratory structural equation modeling (ESEM) to examine the scale’s factor structure. A general community sample of 203 (men = 43, women = 160) between 17 and 63 years completed the QCAE. Although both the five-factor oblique and second order factor models showed good model fit, and clarity in the pattern of factor loadings, in the second-order factor model, none of the primary factors loaded significantly on their respective secondary factors, thereby favoring the five-factor oblique model. The factors in this model were supported in terms of external validity. Despite this, the factor for PRO in this model showed low reliability for meaning interpretation. A revised four-factor oblique model without the PRO factor showed good fit, clarity in the pattern of factor loadings, and reliability and validity for the factors in this model, thereby suggesting this to be the best model to represent ratings on the QCAE.

## Introduction

Although numerous definitions of empathy exist, a common theme in them is that empathy comprises two components: cognitive empathy and affective empathy [1–3].

Cognitive empathy refers to understanding other people’s emotions, whereas affective empathy refers to vicariously experiencing other people’s emotions [4]. While many questionnaires have been developed for measuring empathy [5], two self-report questionnaires that have consider the multidimensional nature of empathy (in particular cognitive and affective empathy) are the Interpersonal Reactivity Index [IRI; 6], and the Questionnaire of Cognitive and Affective Empathy [QCAE; 4]. While both the IRI and QCAE are popular measures of empathy, recently the construct validity of the IRI (in particular its Personal Distress scale) has been seriously questioned [4, 7, 8]. Given this, and the QCAE was developed from the IRI and other measures of empathy, it is conceivable that the QCAE may be seen as a more preferable measure of empathy than the IRI. However, we believe that the factor structure of QCAE is yet to be clearly established. In line with this, the aim of the current study was to provide a more comprehensive evaluation of the factor structure of this measure, using methods (e.g., item level analysis, and exploratory structural analysis with target rotation) not used previously, and also the reliability and external validity of the ensuing preferred QCAE factor model.

### Initial scale development and validation study

In the initial development and validation study of the QCAE, Reniers et al. [4] applied principal components analysis (PCA) on 65 items related to empathy that was obtained from a range of previously validated empathy questionnaires, such as Baron-Cohen and Wheelwright’s [9] Empathy Quotient. The findings in the PCA were interpreted as supportive of a five-factor oblique model. The five factors were perspective taking (PT; measuring the capability to put oneself in another person’s shoes), online simulation (OS; measuring attempts to put oneself in another person’s position by imagining what that person is feeling and is likely to be used for future intentions), emotion contagion (EC; measuring the automatic mirroring of other’s feelings), proximal responsivity (PRO; measuring the emotional responsiveness to the feelings of others who are close within the social or affective subject’s context), and peripheral responsivity (PER; similar to POR, but its context is detached, such as experiencing empathy with protagonists in a film or a novel) [4]. In all, these factors comprised 31 (all items having factor loadings of > .40) of the original 65 items. In a separate sample, independent clusters confirmatory factor analysis (ICM-CFA), with maximum likelihood estimation, supported adequately the five-factor oblique model and also a hierarchical structure in which the PT and OS factors loaded on a second-order factor called cognitive empathy; and the EC, PRO and PRE factors loaded on another second-order factor called affective empathy [4]. It is to be noted however that in the CFA, item parcels (sum scores involving 2 and 3 items) rather than scores for the individual items were used as observed indicators. Reniers et al. [4] also reported good reliability and validity for the five QCAE factors. The internal consistency Cronbach alpha values for PT, EC, OS, PER and PRO were .85, .72, .83, .65, and .70, respectively. In relation to the validity, the five QCAE factors showed the expected theoretical relations with empathic anger, impulsivity, aggression, psychopathy, and Machiavellianism. Additionally, the strength of the relations differed across cognitive empathy and affective empathy. Cognitive empathy showed stronger negative relationships with dysfunctional impulsivity, and secondary psychopathy, whereas affective empathy showed stronger relationships with empathic anger and expressive aggression. There was also support for the convergent validity in that the QCAE cognitive and affective empathy scores showed strong positive correlations with the cognitive and affective empathy scores of the Basic Empathy Scale [BES; 10]. Based on all these findings the 31-items version of the QCAE was adopted as the final version of the QCAE, with the five-factor oblique model (the primary factors being PT, OS, EC, PRO and PER), and the second order model with two secondary factors (cognitive empathy and affective empathy) and five primary factors (PT, OS, EC, PRO and PER) as appropriate measurement models.

### Other factor analysis studies of the QCEA: Findings, problems, and problems and solutions

Since Reniers et al.’s [4] initial scale development and validation study, a number of studies have investigated the factor structure of the QCEA using PCA [e.g., 11] and ICM-CFA [5, 11–15]. Virtual all the ICM-CFA studies used the same sets of parcels as used by Reniers et al. [4]. Keeping this in mind, the findings across these studies reported adequate [11, 13, 15] or good [5, 6] support for the five-factor oblique and the second-order factor models, with the five-factor oblique model proposed to be a slightly better model [15]. No support was found for a one-factor model (all items loading on a single factor) or a two-factor model (all the PT and OS items loading on a first order factor model called cognitive affect model, and all EC, PRP, PER items loading on a second first order factor called affective empathy; [5, 15]). The Queirós et al. [12] study also examined the five-factor and second order factor models at the item level. In contrast to the findings involving parcels, they found adequate, but mixed, support for both these models. A study by Horan et al. [14] found that the four PER items were uncorrelated with each other and with any of the remaining 27 QCEA items, an ICM-CFA conducted without the four PER items indicated support for a two-factor model. Although these factors were referred to as cognitive and affective empathy, they were composed of many items that were different from those in the model proposed by Reniers et al. [4]. The PCA study by Liang et al. [11] that used the 31 items as indicators found support for a four-factor model. The factors were PT, OS, PER, and combined EC + PRO. However, there were some differences in the item contents of the factors, compared to that reported by Reniers et al. [4]. These past studies have also provided support for the external validity of the QCEA.

Taken together, although past studies provide reasonable support for the theorized five-factor model and the second order factor models for the QCEA, the support is not consistent and robust and is on shaky grounds. Thus, further research is needed in this area. In this respect, we wish to argue that future studies must at least give serious considerations to two issues if the goal is to provide a better and thorough understanding of the factor structure of the QCEA. The first issue relates to the use of parcels as indicators in the CFA models tested in past studies. Although parceling has some advantages, such as reducing the amount of measurement error and non-normality in the indicators [16–18], it will also hide many forms of model misspecification, thereby producing biased parameter estimates, especially if the measure in question is multidimensional [18–20]. According to Marsh et al. [20], parceling should never be used if there is no clear established support for the model at the item level. They have recommended using item-level data to evaluate latent variable models. Indeed, past research studies focusing on the factor structure of the QCAE have also urged that future studies in this area conduct CFA at the item level [5, 15].

The second issue relates to using ICM-CFA for testing the factor structure of the QCEA, as done in all previous CFA studies in this area. In the ICM-CFA approach, all items (or parcels) are loaded only to their designated factors and all cross-loadings are restricted to zero [21, 22]. This requirement is considered highly constraining as items are rarely pure indicators of a construct, especially in multidimensional measures, with closely related factors (as is the case with the QCEA). Consequently, they will have variances that will be shared with other items designated for other factors [23], which is indicative of the need to model cross-loadings. Related to this, there is evidence that failure to model even small cross-loadings (as small as 0.100) when present could lead to inflated bias parameter estimates (see review by Asparouhov et al. [24]).

With reference to the QCEA, in the initial scale development and validation study, virtually all items selected by Reniers et al. [4] from the PCA of the initial version of the QCEA to form the final version of the QCEA showed cross loadings ranging from 0.100 to .406 (see Table 2 in Reniers et al. [4]). In all, 14 items cross-loaded on one factor, 9 items cross-loaded on 2 factors, and five items cross-loaded on 3 factors with values of .10 or more. Only 3 items did not cross-load at this level. Additionally, the cross-loadings were most pronounced for the PRO factor, with all four items showing cross-loadings of .10 or more. For this level of cross-loading, items 7, 10 (one at .391), 12 (one at .406), and 23 had 1, 2, 2, and 3 cross-loadings, respectively. As this would suggest that the PRO factor may be especially poorly defined, its inclusion in the QCEA is questionable.

The level of cross-loading found for the QCEA means that when ICM-CFA is used to model QCEA ratings they may show bias estimates, and consequently poor fit, even when this may not be the case. Additionally, this highlights the need to model QCEA ratings taking into account these cross-loadings (albeit low) for a better understanding of its factor structure and is most likely an appropriate explanation for the inconsistent support for the theorized five-factor model and the second order factor models for the QCEA.

To remove the limitations of CFA approach (i.e., restricting all cross-loading to zero), the exploratory structural equation modeling (ESEM) with target rotation procedure has been developed [25, 26]. ESEM with target rotation combines the advantages of the EFA approach (allowing cross-loadings) with the CFA approach (model-based and testing *a priori* defined structure). S2 Fig (Model 3; see S2 Fig) demonstrates the schematic representation of the ESEM with a target rotation model as applied to QCEA. As shown, the targeted symptoms present on their own factors as well as the non-targeted factors. These cross-loading values are set, but not forced, to values close to zero. Available evidence has indicated the advantages of the ESEM with target rotation over other procedures [i.e., CFA and EFA; 26, 27]. A feature of the ESEM approach that is of particular relevance to QCEA is that it can be used to examine if there is justification for using parcels (instead of individual items) in the ICM-CFA model. In this respect, when the fit for the CFA and ESEM models are acceptable and equally good, the use of parcels in the CFA is justified. In contrast, if the fit for the ESEM model is superior to the fit of the CFA model, the use of parcels is not justified. Thus, the application of the ESEM approach will not only provide a better approach for understanding the factor structure of the QCEA, but it will also allow us to ascertain if the use of parcels for examining the factor structure of the QCAE (as done in past CFA studies) has justification. To our knowledge, the ESEM with target rotation approach has not been applied in any past literature involving the QCEA, although, as mentioned earlier, there clear advantages in doing so.

### Additional requirements for the creditability of the factors in a model

For the acceptance of any model, including an ESEM model, it is necessary that the factors in the model are clearly defined, and have good reliability and external validity. The clarity of the factors in the model can be ascertained by examining the significance and salience of factor loadings, and cross-loadings in the case of an ESEM model [23, 28]. Although the internal consistency reliability of the items in a factor is often reported in terms of the Cronbach alpha coefficient, this coefficient is biased [29]. A more accurate estimation of internal consistency reliability is omega [ω; 30]. Omega is an index of model-based internal consistency reliability. The ω values are required to be at a minimum of .50, with a value of at least .75 for meaningful interpretation of a scale [31]. The external validity of factors in a model is usually established by examining if the factors are associated in theoretically and/or empirically meaningful ways with appropriate external variables. As reviewed earlier, for the five-factor oblique model, past studies have shown support for the external validities of the five primary factors in the QCAE.

### Testing the external validity of the QCEA factors using shame, guilt, cognitive reappraisal, and emotion suppression

Ideally, as used in a number of past studies, other measures of empathy would be preferred for testing the external validity of the QCEA factors. However as already noted, apart from the IRI, the other existing empathy measures do not reflect a multidimensional view of empathy (i.e., lack separate scales for cognitive and affective empathy). Although the IRI has separate scales for cognitive and affective empathy, the construct validity of the IRI (in particular its Personal Distress scale) has been seriously questioned. Given this, and as the data in the current study were collected for a primary project that focused on the relationships of emotion regulation, empathy, shame, guilt, and social anxiety, we evaluated the external validity of the QCEA factors using measures of shame, guilt, and emotional regulation (cognitive reappraisal, and emotion suppression). We explain below the justification and rationale for this.

Although past QCAE factor analysis studies have not used shame, guilt, cognitive reappraisal, and emotion suppression to test external validity, these variables can be considered suitable for this purpose. Shame and guilt are considered self-conscious emotions, expressed over concerns from the effects of one’s transgressing behavior on others [32]. Shame refers to a negative evaluation of the self as unworthy, whereas guilt involves a negative evaluation of the transgressing behavior. Therefore, shame involves avoidance and concealing responses that are considered maladaptive; and guilt involves approach and reparation responses that are considered adaptive [33]. For guilt, existing data show that it is associated positively with empathy [32, 34–37]. A recent study reported that both affective empathy and cognitive empathy were associated positively with guilt [38]. For shame, the findings have been mixed, with some studies reporting positive association [34, 37], and other studies reporting negative [32] or no [35, 36] association with empathy. Gambin and Sharpe [38] reported that shame was associated positively with affective empathy and showed no association with cognitive empathy. Thus, based on past studies it can be speculated that both cognitive and affective empathy will be associated with guilt. Although the findings from past studies are mixed, the findings reported by Gambin and Sharpe [38] raises the possibility that shame will be associated positively with affective empathy and show no association with cognitive empathy.

Emotional regulation refers to one’s ability to influence one’s own emotional responses. Cognitive reappraisal and emotion suppression are two broad categories of emotional regulation strategies [39]. Cognitive reappraisal refers to strategies aimed at altering the way one is responding emotionally to the emotion-eliciting stimulus; whereas emotion suppression refers to efforts to inhibit behaviors associated with the emotion being experienced [39, 40]. As emotional regulation is viewed as a macro component of empathy, especially in its development [41], it can be considered relevant for examining the external validity of the QCAE in that there will be a negative association between these constructs [5]. Emotional regulation has been used in a past study to validity the QCAE [e.g., 5].

Existing data show that cognitive reappraisal is more adaptive than emotion suppression [39]. Indeed, emotion suppression may be dysfunctional [42, 43], and associated with negative social outcomes [44, 45]. Past studies have shown positive associations for cognitive reappraisal with cognitive empathy [46–49]; and also, no association [46, 49] or negative association [47, 48] with affective empathy. In the case of emotion suppression, past findings have shown that it has either no association [47, 48] or positive association [46] with cognitive empathy. Also, it has no association [46, 47] or negative association [48] with affective empathy. Thus, based on past findings it could be speculated that cognitive reappraisal will be associated positively with cognitive empathy, and that it will have negative or no association with affective empathy. Also, emotion suppression will have either positive or no association with cognitive empathy and that it will have negative or no association with affective empathy.

### Aims of this study

Given existing limitations and omissions in the literature on the factor structure of the QCEA, the major aim of the current study was to examine support for the proposed five-factor oblique and second order factor models of the QCEA using ICM-CFA and ESEM at the item levels. The CFA models tested are shown in S1 Fig, and the ESEM models tested are shown in S2 Fig. We also computed reliabilities in terms of omega [ω; 30] for the factors in the potentially optimum model(s). Continuing, we examined support for the external validities of the factors in potentially optimum model(s). This was done by regressing the scores for shame, guilt, cognitive reappraisal, and emotion suppression on the factors in these models. We speculated that the ESEM versions of the five-factor oblique and second order factor models of the QCEA would indicate a better and good fit than the ICM-CFA version of these models. Also, the factors in these models would have good omega reliabilities values and will be supported in terms of external validity.

## Method

### Participants

A total of 203 (men = 43, women = 160) adult individuals (age ranging from 18 to 63 years) from the general community participated in the study (see S1 Table in S1 File). The mean age (*SD*) of all participants together was 30.99 years (11.79). The mean age (*SD*) of men and women were 26.65 years (7.79) and 32.15 years (12.42), respectively. Although the gender groups differed significantly for age, *t* (101) = 2.76, *p* < .01, the effect size of this difference was low (cohen’s d = .165). It is noted that the maximum sampling error for a sample of the size of 203 is minus/plus 6.88 for a 95% confidence interval (z = 1.96). S1 Table in S1 File also shows other background information of the participants in the study. As shown, the majority of participants were employed or had completed higher education (technical or university education) and were in some sort of relationship.

In terms of sample size, for this study, we used the a-priori sample size calculator software developed by Soper [50] to ascertain the minimum sample size for the study. For this software, we set the alpha probability level at 0.05, desired statistical power at .80, number of latent variables at 5 (corresponding to the five-factor CFA model tested), number of observed variables at 31 (corresponding to the total number of items in the QCAE), and effect size at .3 (medium effect size). The calculator recommended a minimum sample size of 233. As this requirement is very close to the 203 participants used in the study, adequate power for the study can be assumed.

### Measures

All participants completed a demographic sheet that asked for their age, gender, education, employment, and relationship status. The following questionnaires were completed by participants.

#### Questionnaire of Cognitive and Affective Empathy [QCAE; 4]

Cognitive empathy (19 items) and affective empathy (12 items) were measured using the Questionnaire of Cognitive and Affective Empathy [QCAE; 4]. The Cognitive Empathy scale has subscales for PT (“I am good at predicting what someone will do”), and OS (“Before criticizing somebody, I try to imagine how I would feel if I was in their place”). The affective empathy scale has subscales for EC (“It worries me when others are worrying and panicky”), PRO (“I get very upset when I see someone cry”), and PER (“I often get deeply involved with the feelings of a character in a film, play, or novel”). Each item is rated on a 4-point Likert scale: 1 (strongly agree), 2 (slightly agree), 3 (slightly disagree), and 4 (strongly disagree), with higher scores indicating higher levels of empathy. In the initial scale development and validation study, Reniers et al. [4], reported strong internal, convergent and external validity of the QCAE. The internal consistency alpha values for PT, EC, OS, PER and PRO in the study were .85, .72, .83, .65, and .70, respectively. The internal consistency of Cronbach’s alpha values for cognitive empathy and affective empathy in the current study was 0.85, and 0.86, respectively. They were 90, .86, .73, .68, and .60 for PT, EC, OS, PER and PRO, respectively.

#### Emotion Regulation Questionnaire [ERQ; 44]

The ERQ (10 items) is a self-rating scale that measures cognitive reappraisal (6 items, an example being “To feel less negative emotion, I change my thoughts”); and emotion suppression (4 items, an example being “When feeling positive emotions, I’m careful not to express them”). Each item is rated on a Likert scale ranging from 1 (strongly disagree) to 7 (strongly agree), with higher scores reflecting more use of the strategies. It has sound psychometric properties [44]. The internal consistency (Cronbach’s alpha) for the cognitive reappraisal and emotion suppression subscales in the current study were .78 and .74.

#### Test of Self-Conscious Affect-3 [TOSCA-3; 51]

The TOSCA-3 includes 16 scenarios that can capture how individuals respond to moral dilemmas. An example of a scenario is “You are driving down the road and you hit a small animal. (a) You would think the animal shouldn’t have been on the road. (b) You would think: ‘I’m terrible.’ (c) You would feel: ‘Well, it’s an accident.’ (d) You’d feel bad you hadn’t been more alert driving down the road.” Respondents rate their responses on a 5-point scale ranging from 1 (“not likely”) to 5 (“very likely”) on scales that include shame (16 items), and guilt (16 items). The scores on these scales were used in the current study. Both the shame and guilt subscales have been shown to have good reliability and validity [51]. The Cronbach’s alpha values for the subscales in the current study were .74 and .82.

### Procedure

Details of the study, approved by the Federation University Australia Human Research Ethics Committee, were advertised widely on various on-line platforms used by the general community, and on various community noticeboards. Participants completed the questionnaires either online (51%) or via hard copies. In the case of hard copies, potential participants were given the set of questionnaires in envelopes by students completing an honours psychology degree. Each envelope included an informed consent form and a prepaid reply envelope. For online completion, respondents had to click the survey link that led them to the same set of questionnaires. Proceeding with the survey was taken as providing informed consent.

### Statistical analyses

M*plus* Version 7.3 [52] was used to conduct all the CFA and ESEM analyses. WLSMV extraction was used for these analyses as the scores for the QCAE ratings were ordered-categorical scores [53]. The ESEM models were conducted using geomin (oblique) rotation. To specify the higher-order ESEM model, we used the ESEM-within-CFA method proposed by Morin and Asparouhov [54].

To establish the best model, we used a four-step sequential, which we called stepwise algorithm for model selection (SAMS) procedure. These steps involved four criteria: (i) model fit criterion; (ii) clarity criterion, (iii) reliability criterion and (iv) validity criterion. Step 1 (model fit criterion) compared and examined the global fit values of all models tested. As large samples will inflate χ^2^ values, model fit was evaluated using root mean squared error of approximation (RMSEA), comparative fit index (CFI), Tucker Lewis index (TLI), and the Weighted Root Mean Square Residual (WRMR). A model was deemed a potentially good model if all four of these approximate fit indices indicated a good fit. According to Hu and Bentler [55], RMSEA, values < .06 = good fit, < .08 = acceptable fit, and > .08 to .10 = marginal fit. For CFI and TLI, values ≥.95 = good fit, and ≥ .90 = acceptable fit. For the WRMR, values < 1 suggest a good model fit [56]. Where needed, the difference in the fit of nested models was examined using differences in RMSEA (≥ 0.015) and CFI (≥ 0.010) values [57, 58]. We selected good fitting models, regardless of whether they differed from each other.

In step 2, (clarity criterion) all models selected as potentially good were examining for the salience of symptom factor loadings and cross-loadings (in ESEM). As in the original study by Reniers et al. [4], an item was considered salient if it had a loading of >.40. Factors with more salient loadings of designated symptoms and fewer salient loadings of non-designated symptoms were considered to be better defined. As this may leave two or more models equally supported, in steps 3 and 4 we examined the reliabilities and external validities of the factors in these equally supported models.

In step 3 (reliability criterion), omega (ω) values for the factors were assessed [30]. Relative to coefficient alpha, the ω provides a model based (and better) measure of the internal consistency of a factor [29]. As explained earlier, ω values are required to be at least .50, and with a value of at least .75 for meaningful interpretation of a scale [31]. In step 4 (validity criterion), to test the external and differential validities of the factors in potentially good models, the ERQ scores for cognitive reappraisal and emotion suppression; and the TOSCA-3 scores for shame and guilt were regressed (separately) on all the factors in the relevant QCEA model. Support for the external validity of the factors was inferred from theoretical and/or empirical meaning and significant positive associations with cognitive reappraisal, emotion suppression, shame and guilt. Differences in the patterns of significant positive associations between the factors with the external variables were interpreted as evidence of differential validity of the QCAE factors. The potentially good model with better support for reliabilities and validities of the factors was considered the most preferred optimum model.

## Results

### Comparison of global fit and correlations between latent factors (SAMS step 1 model fit criterion)

Table 1 provides the global fit values for all the QCEA models that were tested in the study. As shown, all the fit values for both the CFA models (Model 1 and 2) showed poor fit. In contrast, the ESEM models (Model 3 and 4) showed a good fit. Models 3 and 4 did not differ from each other (ΔCFI < .01). These findings indicate that Models 3 and 4 can be considered as potentially good models for representing the ratings of the QCEA items.

**Table 1:** Fit values for the QCAE CFA and ESEM models tested in the study.

Model	*χ* ^2^	*df*	RMSEA (90% CI)	CFI	TLI	WRMR
CFI						
M1: CFA-5	946.985***	424	.078 (.071-.085)	.888	.877	1.412
M2: H-CFA-5	959.527***	428	.078 (.072-.085)	.886	.876	1.461
ESEM						
M3: ESEM-5	507.391***	320	.054 (.045-.062)	.960	.942	.666
M4: H-ESEM-5	501.102***	324	.052 (.043-.061)	.962	.946	.666

*Note*. M1: CFA-5 = correlated five-factor model; M2: H-CFA-5 = Higher-order five-factor model; M3: ESEM-5 = five-factor ESEM; M6: H-ESEM-4 = Higher-order ESEM with five first-order factors; χ2 = chi-square fit statistic; *df* = degrees of freedom; CFI = comparative Fit Index; TLI = Tucker-Lewis Index; RMSEA = root mean square error of approximation and its 90% confidence interval(CI); WRMR = Weighted Root Mean Square Residual.

***p < .001.

### Magnitude and pattern of factor loadings in the ESEM model with two specific factors (SAMS step 2 clarity criterion)

Table 2 details the factor loadings for the ESEM models with five primary factors (Model 3) and the second-order factor model (Model 4) that passed the SAMS step 1 criterion (good model fit). As shown in the table, the pattern of loadings and cross-loadings were highly comparable for the two models. For both models, there were four items that did not load saliently (> .40) on the designated factors. They were item numbers 31 (an OS item), 17 (a PER item), and 7 and 10 (both PRO items). There were three salient cross-loadings in the five-factor oblique model, and four salient cross-loadings in the second order factor model. Both models had salient cross-loadings for item numbers 23 (a PRO item cross-loading on PT), and 5 and 10 (both PRO items cross-loading on EC). The additional cross-loading for the second order factor model was item number 21 (a PT item cross-loading on the PRO factor). Thus, the PRO factor was not clearly defined, as it has only two of four items that loaded saliently, and the two non-salient items cross-loading saliently on the EC factor.

**Table 2:** Item factor loadings, cross-loadings and factor reliabilities for the QCAE ESEM-5 and H-ESEM-5 models.

Item	M3: ESEM-5					M4: H-ESEM-5				
Item Factor Loadings and Cross-Loadings										
# (in QCAE). Brief description	PT	OS	EC	PRE	PRO	PT	OS	EC	PRE	PRO
15. enter a conversation.	**.72**	.03	-.14	.09	.10	**.71**	.03	-.14	.08	.18
16. says one but means another	**.79**	-.03	-.13	.09	.00	**.78**	-.03	-.14	.09	.08
19. predicting how someone feel	**.65**	.25	.03	.03	-.05	**.64**	.24	.02	.03	.05
20. feeling awkward in group	**.63**	.13	-.09	.08	.13	**.62**	.13	-.10	.08	.20
21. understanding feeling/thinking	**.67**	.05	-.05	.09	.37	**.67**	.06	-.06	.08	.43
22. interested or bored	**.72**	-.08	-.14	.06	.22	**.71**	-.07	-.14	.05	.29
24. sense if I am intruding	**.54**	.06	.00	-.03	.24	**.54**	.06	-.01	-.04	.32
25. person might want to talk	**.82**	.03	.11	-.06	-.15	**.80**	.02	.10	-.06	-.04
26. masking their true emotion	**.95**	-.10	.17	-.14	-.17	**.94**	-.11	.16	-.15	-.05
27. predicting what someone do	**.87**	-.08	.14	-.13	-.34	**.85**	-.09	.14	-.12	-.23
1. difficult to see from other’s view	-.05	**.68**	-.26	.12	.07	-.04	**.67**	-.27	.12	.10
3. everybody’s side of disagreement	.07	**.79**	.02	-.22	-.03	.08	**.78**	.01	-.21	.06
4. how things look from their perspective	.13	**.72**	.08	.02	-.14	.13	**.71**	.07	.03	-.05
5. “put myself in his shoes”	-.07	**.92**	.03	.10	-.33	-.08	**.90**	.02	.12	-.26
6. imagine feeling if I was in their place	.00	**.83**	.00	.02	-.14	.00	**.82**	-.01	.03	-.06
18. easy to put in somebody else’s shoes	.12	**.71**	-.19	.24	.02	.11	**.71**	-.20	.25	.08
28. appreciate other person’s viewpoint	.11	**.55**	-.02	-.13	.16	.12	**.55**	-.03	-.14	.23
30. consider the other fellow’s feelings	-.14	**.75**	.14	-.17	.39	-.12	**.75**	.13	-.18	.46
31. how my friends will react to it	.05	**.36**	.26	-.04	.23	.05	**.36**	.26	-.05	.29
8. get nervous when others nervous	-.01	.08	**.78**	.06	-.02	-.03	.08	**.77**	.07	.05
9. people influence on my mood	.00	.01	**.63**	.06	.08	-.01	.00	**.62**	.07	.13
13. happy in a cheerful group	.08	-.08	**.48**	.11	.25	.08	-.08	**.47**	.10	.29
14. worries me when others are worrying	.03	-.05	**.61**	.14	.21	.02	-.05	**.61**	.14	.25
2. usually objective when watch a film	.01	-.13	-.01	**.78**	-.02	-.02	-.13	-.01	**.80**	-.06
11. involved with feelings of film character	.01	.05	.26	**.71**	-.02	-.02	.05	.25	**.73**	-.02
17. hard to see why people get upset	.05	.33	-.15	**.29**	.15	.04	.32	-.15	**.28**	.18
29. stay emotionally detached	.00	-.03	-.09	**.84**	-.01	-.04	-.03	-.09	**.86**	-.05
7. emotionally involved with problems	.07	.04	.51	.21	**.03**	.06	.03	.50	.21	**.07**
10. affects me when friends seems upset	.11	.19	.51	.12	**.25**	.11	.18	.50	.12	**.32**
12. upset when I see someone cry	.00	-.02	.39	.19	**.40**	.00	-.01	.38	.18	**.43**
23. friends talk to me about problems	.47	.13	-.09	.03	**.41**	.46	.15	-.10	.01	**.49**
Degree of clarity of factor loadings										
Maximum number of salient target loadings	10	9	4	4	4	10	9	4	4	4
Observed number of salient target loadings	10	8	4	3	2	10	8	4	3	2
Maximum number of non-target loadings	21	22	27	27	27	21	22	27	27	27
Observed number of non-target loadings	1	0	2	0	0	1	0	2	0	1
Reliability										
Omega	.92	.90	.72	.77	.25	.92	.90	.72	.77	.25

ESEM-5 = five-factor ESEM; M4: H-ESEM-5 = Higher-order ESEM with five first-order factors; PT = Perspective taking; OS = Online simulation; EC = Emotion contagion; PRE = Peripheral responsivity; PRO = Proximal responsivity.

### Correlations of the factors in the QCAE ESEM-5 and standardized path coefficient in second-order factor model H-ESEM-5 models

Table 3 provides the inter-correlations among the factors in the five-factor model, and the standardized path coefficients for the relations between the second-order factors and the primary factors in the second-order factor model. As will be noticed, in the five-factor oblique model, with the exception of the correlations of PT and OS with EC, all other correlations were significant. The correlations for PT with OS were moderate (*r* = .61), and all the other inter-correlations, including the inter-correlations between FC, PER and POR that belong to the affective empathy higher-order factor, were low, ranging from .24 to 14. Thus, the inter-correlation was not compatible with an affective empathy higher-order factor.

**Table 3:** Correlations for the factors in the QCAE ESEM-5 and standardized path coefficient in second-order factor model H-ESEM-5 models.

	PT	OS	EC	PER	PRO
Correlations					
Perspective taking (PT)	1				
Online simulation (OS)	.61***	1			
Emotion contagion (EC)	.08	.04	1		
Peripheral responsivity (PER)	.24***	.16*	.24***	1	
Proximal responsivity (PRO)	,24***	.19***	.14**	.21***	1
Standardized path coefficient in second-order factor model					
Cognitive empathy	1.00	.60			
Affective empathy			.25	.87	.31

In the second-order factor model, the path coefficients for all the primary factors on their respectively secondary factors were not-significant, thereby indicating insufficient support for grouping the primary factors into separate cognitive and affective empathy higher order factors. Overall, therefore, the findings indicate more support for the five-factor oblique model over the second-order factor model. Given this, we examined the reliabilities and external validities of the factors in the five-factor oblique model.

### Reliabilities of the factors in the five-factor ESEM oblique model (SAMS step 3 reliability criterion)

As shown in Table 2, the omega values were .92, .90, .72, .77 and .25 for PT, OS, EC, PER and PRO, respectively. As Reise et al. [31] have proposed that omega values need to be at least .50 for meaningful interpretation of a scale, it follows that while the scores for the factors for PT, OS, EC, and PER can be meaningfully interpreted, the score for the PRO factor cannot be meaningfully interpreted.

### Validities of the factors in the five-factor ESEM oblique model (SAMS step 4 validity criterion)

Table 4 shows the path coefficients in which shame and guilt; and cognitive reappraisal and emotion suppression were regressed on the factors in the five-factor oblique model. As shown for the regression analyses involving shame and guilt, shame was associated significantly and negatively with PT, and significantly and positively with EC. In contrast, guilt was associated significantly and positively with EC and PRO. However, the association with PRO may be meaningless given its low reliability. For the regression analyses involving cognitive reappraisal and emotion suppression, cognitive reappraisal was associated significantly and negatively with EC, and significantly and positively with OS and PER. Emotion suppression showed no association with any of the factors in the model. On the basis of past studies (reviewed in the introduction), the findings in our study can be interpreted as supportive of the external and differential validities of the PT, OS, EC, and PER factors in the five-factor oblique model.

**Table 4:** Standardized path coefficients for the predictions of the dimensions of shame and guilt, and cognitive reappraisal & emotion suppression by the dimensions in the QCAE ESEM-5 model.

	TOSCA-3		ERQ	
	Shame	Guilt	Cognitive reappraisal	Emotion suppression
Perspective taking	-.34*	-.15	-.04	.09
Online simulation	.04	.31***	.34**	-.04
Emotion contagion	.37***	-.00	-.41***	.07
Peripheral responsivity	.10	,01	.23*	-.17
Proximal responsivity	.27	.24*	.01	-.08

*Note*. TOSCA-3 = Test of Self-Conscious Affect-3; ERQ = Emotion Regulation Questionnaire; QCAE = Questionnaire of Cognitive and Affective Empathy; ESEM-5 = five-factor ESEM.

### Selection of a preferred model for the QCAE

Although the SAMS steps can be understood as providing support for the five-factor oblique model, this model has limitations in that the PRO factor in this model lacked sufficient reliability. Given this, it is conceivable that a more preferential model would be one that does not include the PRO items/factor. The fit values for a four-factor oblique model (with primary factors for PT, OS, EC, and PER), without the PRO items, were WLSMVχ^2^ (*DF* = 245) = 460.24, *p* < .001; RMSEA = .065 (90% CI = .055–.074); CFI = .951; TLI = 0.931; and WRMR = .750. The values can be interpreted as indicating a good model-data fit. The pattern of factor loading for this model, and the reliability and external validity of four factors in this model, were highly comparable to those reported for these factors in the five-factor oblique model, and for this reason not provided here separately.

## Discussion

The study used ICM-CFA and ESEM with target rotation to examine support for the proposed five-factor oblique (primary factors being PT, OS, EC, PER and PRO), and the second order factor (higher-order factors being cognitive and affective empathy, and primary factors being PT, OS, EC, PER and PRO) models of the QCAE. Both models showed poor fit when ICM-CFA was applied. In contrast, both models showed a good fit when ESEM with target rotation was applied. For both the ESEM models, there were only four items (the same items in both models) that did not load saliently (> .40) on the designated factors. In relation to cross-loadings, there were only three salient cross-loadings in the five-factor oblique model and only four salient cross-loadings in the second order factor model. The PRO factor in both models was not clearly defined. Thus, the pattern of loadings and cross-loadings were highly comparable for the two models, and with the exception of the PRO factor, all the other factors can be considered to be well defined. Although both the five-factor oblique model and the second order factor model showed potential to be appropriate models for the QCAE, the higher-order factors model was problematic in that the path coefficients for all the primary factors on their respectively secondary factors were not-significant. Thus, between the five-factor oblique and the second order factor models, the five-factor oblique model demonstrated more acceptability, whereas the second order factor model proved to be unacceptable. Although there was support for the external and differential validities of all the five factors in the ESEM five-factor oblique model, for the factors in this model, the factor for PT, OS, EC, and PER showed good internal consistency reliability, whereas the factor for PRO showed insufficient reliability for its meaningful interpretation. In view of this, and also as the PRO factor was not clearly defined, this factor in the QCAE could be considered not appropriate.

The lack of clarity and reliability in the PRO factor is sufficient reason to drop the PRO items and the PRO factor as part of the QCAE. If the PRO items and factors are removed from the QCAE a four-factor oblique model (with primary factors for PT, OS, EC, and PER) can be speculated for the QCAE. For this model, ESEM with target rotation indicated good model-data fit, with the pattern of factor loadings and cross-loadings, reliability, and external validity comparable to those reported for the four factors in the five-factor oblique model. Consequently, we are proposing that the most appropriate QCAE factor model is a first-order four-factor oblique model with primary factors for PT, OS, EC, and PER, with the items in these factors the same as that proposed for these factors in the initial study by Reniers et al. [4]. However, there is one problem in this model that is worth noting. In this model, OS item numbers 31 and PER item number 17 did not load saliently on their designated factors. The loading for item numbers 31 and 17 on their designated factors were .36 and .29, respectively. Given that some researchers consider loadings of ≥.30 as salient [59], it follows that item 17 is particularly problematic. Indeed, the study by Queirós et al. [12] also found extremely low loading for item number 17, and they have suggested that this item be removed from the QCAE.

To the extent that the first-order four-factor oblique model proposed for the QCAE evolved from an examination of the five-factor oblique model proposed originally by Reniers et al. [4] as the theoretical model for the QCAE, the findings are comparable to past studies in this area which examined the five-factor structure of the QCAE. The findings in these studies, based on ICM-CFA, reported either adequate [4, 11, 13, 15] or good [5, 12] fit for the five-factor oblique model.

### Differences of the present study compared to past findings

Despite this comparability, there are two major areas that make the current study novel and distinct from previous studies. First, with the exception of the study by Queirós et al. [12], past studies in this area have used parcels as indicators in their models. In contrast, the current study used the individual items as indicators in the models. Compared to parcels, items are considered more appropriate as parcels could hide many forms of model misspecification, thereby producing biased parameter estimates [18–20]. Indeed, Marsh et al. [20] have argued that parceling should never be used and have recommended item-level data to evaluate latent variable models, especially if there is no prior support for the model at the item level, and if the measure in question is multidimensional, as is the case with the QCAE. For item-level analysis, the study by Queirós et al. [12], which involved a community sample in Portugal, reported acceptable fit in terms of CFI and TFI values (CFI = .91 and TLI = .90), and poor fit in terms of the RMSEA and SRMR values (RMSEA = .10; SRMR = .09) for the CFA five-factor oblique model. Therefore, there was mixed support for this model. In contrast to that study, the CFA findings in the current study showed poor fit for this model, with fit in terms of all four fit indices: CFI = .888; TLI = .877; RMSEA = .078; WRMR (an index that is appropriate to the SRMR when WLSMV extraction is used) = 1.412. Taken together, at best, the findings from both these ICM-CFA findings suggest only adequate marginal mixed fit. This is not an impressive finding for guiding the way the ratings of the QCAE have to be used.

A second major difference between the current and past studies is that, unlike all previous studies that have used ICM-CFA, the current study also used ESEM with target rotation. Unlike ICM-CFA where all cross-loadings are restricted to zero, in the ESEM with target rotation procedure, items load on their own designated factor and also cross-load on all the non-designated factors, with values set, but not forced, to zero [25, 26]. Such a model is considered to be more advanced and closed to the reality of the covariances in the data set [26, 27]. As will be evident by now, in contrast to the findings from the CFA, the findings from the ESEM with target rotation showed good fit for both the five-factor oblique and second-order factor models showed good fit—although the latter model was considered unacceptable as all its five primary factors did not load significantly on their respective higher-order factors. Notwithstanding this, only because we used ESEM with target rotation we were able to conclude clear support for a useable model for the QCAE. This was not the proposed five-factor model, but a closely related four-factor model. This is an impressive finding for guiding the way the ratings of the QCAE has to be used.

At the statistical level, when the fit for the CFA and ESEM models are acceptable and equally good, the use of parcels in the CFA is justified. In contrast, if the fit for the ESEM model is greater than the fit of the CFA model, the use of parcels is not justified [20]. As the fit for the ESEM models was better than the fit of the corresponding CFA model in our study, it can be assumed that the use of parcels for examining the factor structure of the QCAE is not justified [20]. Thus, it can be argued that with the exception of the findings at the item level reported by Queirós et al. [12], the findings in all the previous studies in this area that have reported findings at the parcel level [4, 5, 11–15] are not worthy of serious consideration. What this also means is that all future studies of the factor structure of the QCAE should be conducted at the item level, and not at the parcel level. As this alone is unlikely to provide good fitting models, such studies should also apply ESEM with target rotation. The benefits of these strategies have been clearly demonstrated in the current study.

### Implications

The lack of clarity of the PRO scale indicates that this scale does not adequately measure the empathy facet of proximal responsivity or emotional responsiveness to the feelings of others who are close within the social or affective subject’s context. Relatedly, the PCA study by Liang et al. [11] that used the 31 items as indicators did not find an independent PRO factor. Instead, they found support for a four-factor model, with the factors being PT, OS, PER, and combined EC + PRO. There was also a lack of clarity for the PRO scale in the initial scale development and validation study by Reniers et al. [4]. That study showed substantial cross-loadings with cross-loading being especially high for items 10 (one at .391), and 12 (one at .406). These findings suggest that the PRO scales need considerable revisions if the QCAE is to continue to measure the theoretical model underlying the QCAE. In terms of revisions, our study showed that PRO items 7 and 19 did not load saliently on the PRO factors. Additionally, for both the primarily factor and higher order factor models, PRO items 5, 10 and 23 showed salient cross-loadings. Thus, the findings indicate the need to revise or more likely change all four items in the PRO scale. Notwithstanding the need to revise the PRO scales, and until such time a revised QCAE is produced, we recommend that researchers and clinicians use the QCAE, the scores for the PRO be removed and that only scores for the remaining 27 items be used to derive scores for total empathy, cognitive empathy, PT, PS, affective empathy (based on the items in the EC and PER scales), EC and PER. By way of revision, it is worth noting our previous suggestion for the need to either drop or revised PER item 17 (also recommended by Queirós et al. [12]). The poor construct validity of the PRO scale found in this study, suggests that past findings based on the PRO need to be dismissed or at the very least, interpreted with great caution. Also, this caution needs to be applied for findings based on the total effective empathy scale score and/or the total QCAE empathy score as these scores also include the PRO scale items.

In addition to implications (just described above) for how future studies should examine the factor structure of the QCAE, our findings also have implications for understanding the relations of empathy with emotional regulation, shame, and guilt. Our findings showed that cognitive reappraisal was associated significantly and negatively with EC, and significantly and positively with OS and PER. Emotion suppression showed no association with any of the QCAE factors. Consistent with our findings, past studies have shown positive associations for cognitive reappraisal with cognitive empathy [46–49]; and also, no association [46, 49] with affective empathy. In the case of emotion suppression, past findings have also shown no association [47, 48] with cognitive and affective empathy [46, 47]. Therefore, our findings and these past studies, show that while cognitive reappraisal (or strategies aimed at altering the way one is responding emotionally to the emotion-eliciting stimulus) is not associated with high levels of automatic mirroring of other’s feelings, it is associated with high levels of ability to put oneself in another person’s position by imagining what that person is feeling, and high ability to respond emotionally to the feelings of others in a film or a novel. In contrast, emotion suppression (or efforts to inhibit behaviors associated with the emotion being experienced) has no relation with empathy. These relations also indicate that cognitive empathy is adaptive, as has been proposed in the literature [39].

In relation to shame, our findings showed that shame was associated significantly and negatively with PT, and significantly and positively with EC. Our findings mean that although individuals with high scores for shame are able to automatically mirror the feelings of others, they are less able or willing to put themselves in the shoes of others. This can be interpreted to mean that even when individuals with high shame can understand the feelings of others, they will most likely not respond cognitively and behaviorally in an emotional-congruent manner (i.e., with empathy). Additionally, our findings indicated that guilt was associated significantly and positively with OS and PRO. However, as the PRO scale show poor construct validity, the findings involving PRO can be considered of little relevance, and not worthy of interpretation. Given this, our findings indicate that individuals with high guilt are likely to engage in attempts to put themselves in other people’s position by imagining what ‘they are feeling and is likely to use this in future responses. Thus, they are more likely to consistently respond cognitively and behaviorally in an empathetic manner. Our findings are somewhat consistent with that reported by Gambin and Sharpe [38] who found that shame was associated positively with affective empathy and had no association with cognitive empathy; and that both affective empathy and cognitive empathy were both associated positively with guilt.

### Limitations & further research

A major strength of the current study was that, unlike previous studies, the factor structure was examined at the item level, using ESEM with target rotation. In concluding the findings in this study, found a good fit for a four-factor oblique model, with primary factors for PT, OS, EC, and PER. The factors in both these models were clearly defined in terms of the pattern of factor loadings, reliabilities, and external validities. Despite the strengths in the study, and new and valuable psychometric findings for the QCAE provided in the study, several study limitations have to be considered. First, it is possible that factors such as age, gender, ethnicity, and other psychological problems could influence the ratings of items on the QCAE. Failing to control for these effects (as there is no easy way to do this for CFA and ESEM models) could have confounded the results. Second, the nature of the data collection and the limitations of the ethics approval did not allow for the collection of data about individuals prior to them being invited to participate. Therefore, there is no information about any individuals who knew of the study but did not accept the invitation to participate, and consequently how this affected the results. Third, the study utilized a sample of adults from the general community, therefore it is unknown to what extent these findings might apply to clinically diagnosed adults. Fourth, all data used were collected using self-report questionnaires. It is possible that this method may have influenced the ratings, and consequently, our results may be subject to confounding by common method variance. Finally, the conclusions made in this study are based on a single study. Given these limitations, the results may be seen as preliminary. Notwithstanding these limitations, we argue that the findings of this study do provide the impetus for further research in this area, taking into consideration the methodology used in the current study, and controlling for the limitations highlighted here. Most importantly, we urge that future studies of the factor structure of the QCAE use ESEM with target rotation and examine the items rather than parcels and avoid limiting their analyses to only the ICM-CFA approach.

## Supporting information

S1 File Supplementary material for QCAE paper. (DOCX)

S2 File Dataset. (SAV)

S3 File Supplementary material detailing clinical and methodological significance. (DOCX)

S1 Fig Hypothesized CFA models. (TIF)

S2 Fig Hypothesized ESEM models. (TIF)
