# Supplementary material for: Confirmatory factor analysis and exploratory structural equation modeling of the factor structure of the Questionnaire of Cognitive and Affective Empathy (QCAE)
Source: PLoS One. 2022 Feb 7;17(2):e0261914. doi: 10.1371/journal.pone.0261914 (PMC8820594; doi:10.1371/journal.pone.0261914)
Supplement: S1 File — (DOCX) [file pone.0261914.s001.docx]

Supplementary Material for paper “**Confirmatory Factor Analysis and Exploratory Structural Equation Modeling of the Factor Structure of the Questionnaire of Cognitive and Affective Empathy (QCAE)**”

Supplementary Table S1

*Frequencies and Descriptive Statistics of Background Variables*

| Background variables | Frequency(%)/Descriptive Statistics [Mean (SD)] |
| --- | --- |
| Number of participants | 203 |
| Gender – Male (M)  Female (F) | 43 (21.2%)  160 (78.8%) |
| Age - Mean (SD | 30.99 (11.80) |
| Relationship - In a relationship | 82 (40.4%) |
| Single | 64 (31.5%) |
| Married | 40 (19.7%) |
| Divorced/Separated | 12 (5.9%) |
| Others | 5 (2.5%) |
| Education - Primary School | 3 (1.5%) |
| Secondary School | 57 (28.1%) |
| Trade certificate | 30 (14.8%) |
| University | 105 (51.7%) |
| Others | 8 (3.9%) |
| Employment – Full time | 72 (35.5%) |
| Part time | 73 (36.0%) |
| Not employed | 13 (6.4%) |
| Student | 37 (18.2%) |
| Others | 8 (4.0%) |

**Supplementary Table S2**

*Item Factor Loadings, and Factor Reliabilities, Correlations and Secondary Loading (as Appropriate) for the QCAE ESEM-5 and H-ESEM-5 Models*

| Item | M3: ESEM-5 | | | | | M4: H-ESEM-5 | | | | |
| --- | --- | --- | --- | --- | --- | --- | --- | --- | --- | --- |
| # (in QCAE). Brief description | PT | OS | EC | PRE | PRO | PT | OS | EC | PRE | PRO |
| 15. enter a conversation. | **.72** | .03 | -.14 | .09 | .10 | **.71** | .03 | -.14 | .08 | .18 |
| 16. says one but means another | **.79** | -.03 | -.13 | .09 | .00 | **.78** | -.03 | -.14 | .09 | .08 |
| 19. predicting how someone feel | **.65** | .25 | .03 | .03 | -.05 | **.64** | .24 | .02 | .03 | .05 |
| 20. feeling awkward in group | **.63** | .13 | -.09 | .08 | .13 | **.62** | .13 | -.10 | .08 | .20 |
| 21. understanding feeling/thinking | **.67** | .05 | -.05 | .09 | .37 | **.67** | .06 | -.06 | .08 | .43 |
| 22. interested or bored | **.72** | -.08 | -.14 | .06 | .22 | **.71** | -.07 | -.14 | .05 | .29 |
| 24. sense if I am intruding | **.54** | .06 | .00 | -.03 | .24 | **.54** | .06 | -.01 | -.04 | .32 |
| 25. person might want to talk | **.82** | .03 | .11 | -.06 | -.15 | **.80** | .02 | .10 | -.06 | -.04 |
| 26. masking their true emotion | **.95** | -.10 | .17 | -.14 | -.17 | **.94** | -.11 | .16 | -.15 | -.05 |
| 27. predicting what someone do | **.87** | -.08 | .14 | -.13 | -.34 | **.85** | -.09 | .14 | -.12 | -.23 |
| 1. difficult to see from other’s view | -.05 | **.68** | -.26 | .12 | .07 | -.04 | **.67** | -.27 | .12 | .10 |
| 3.  everybody’s side of disagreement | .07 | **.79** | .02 | -.22 | -.03 | .08 | **.78** | .01 | -.21 | .06 |
| 4. how things look from their perspective | .13 | **.72** | .08 | .02 | -.14 | .13 | **.71** | .07 | .03 | -.05 |
| 5. “put myself in his shoes” | -.07 | **.92** | .03 | .10 | -.33 | -.08 | **.90** | .02 | .12 | -.26 |
| 6.  imagine feeling if I was in their place | .00 | **.83** | .00 | .02 | -.14 | .00 | **.82** | -.01 | .03 | -.06 |
| 18. easy to put in somebody else’s shoes | .12 | **.71** | -.19 | .24 | .02 | .11 | **.71** | -.20 | .25 | .08 |
| 28. appreciate other person’s viewpoint | .11 | **.55** | -.02 | -.13 | .16 | .12 | **.55** | -.03 | -.14 | .23 |
| 30. consider the other fellow’s feelings | -.14 | **.75** | .14 | -.17 | .39 | -.12 | **.75** | .13 | -.18 | .46 |
| 31. how my friends will react to it | .05 | **.36** | .26 | -.04 | .23 | .05 | **.36** | .26 | -.05 | .29 |
| 8.  get nervous when others nervous | -.01 | .08 | **.78** | .06 | -.02 | -.03 | .08 | **.77** | .07 | .05 |
| 9.  people influence on my mood | .00 | .01 | **.63** | .06 | .08 | -.01 | .00 | **.62** | .07 | .13 |
| 13. happy in a cheerful group | .08 | -.08 | **.48** | .11 | .25 | .08 | -.08 | **.47** | .10 | .29 |
| 14. worries me when others are worrying | .03 | -.05 | **.61** | .14 | .21 | .02 | -.05 | **.61** | .14 | .25 |
| 2. usually objective when watch a film | .01 | -.13 | -.01 | **.78** | -.02 | -.02 | -.13 | -.01 | **.80** | -.06 |
| 11. involved with feelings of film character | .01 | .05 | .26 | **.71** | -.02 | -.02 | .05 | .25 | **.73** | -.02 |
| 17. hard to see why people get upset | .05 | .33 | -.15 | **.29** | .15 | .04 | .32 | -.15 | **.28** | .18 |
| 21. stay emotionally detached | .00 | -.03 | -.09 | **.84** | -.01 | -.04 | -.03 | -.09 | **.86** | -.05 |
| 7. emotionally involved with problems | .07 | .04 | .51 | .21 | **.03** | .06 | .03 | .50 | .21 | **.07** |
| 10. affects me when friends seems upset | .11 | .19 | .51 | .12 | **.25** | .11 | .18 | .50 | .12 | **.32** |
| 12. upset when I see someone cry | .00 | -.02 | .39 | .19 | **.40** | .00 | -.01 | .38 | .18 | **.43** |
| 23. friends talk to me about problems | .47 | .13 | -.09 | .03 | **.41** | .46 | .15 | -.10 | .01 | **.49** |
| Degree of clarity of factor loadings | | | | | | | | | | |
| Maximum number of salient target loadings | 10 | 9 | 4 | 4 | 4 | 10 | 9 | 4 | 4 | 4 |
| Observed number of salient target loadings | 10 | 8 | 4 | 3 | 2 | 10 | 8 | 4 | 3 | 2 |
| Maximum number of non-target loadings | 21 | 22 | 27 | 27 | 27 | 21 | 22 | 27 | 27 | 27 |
| Observed number of non-target loadings | 1 | 0 | 2 | 0 | 0 | 1 | 0 | 2 | 0 | 1 |
| Correlations in the six-factor model | | | | | | | | | | |
| Perspective taking (PT) | 1 |  |  |  |  |  |  |  |  |  |
| Online simulation (OS) | .61*** | 1 |  |  |  |  |  |  |  |  |
| Emotion contagion (EC) | .08 | .04 | 1 |  |  |  |  |  |  |  |
| Peripheral responsivity (PER) | .24*** | .16* | .24*** | 1 |  |  |  |  |  |  |
| Proximal responsivity (PRO) | ,24*** | .19*** | .14** | .21*** | 1 |  |  |  |  |  |
| Standardized path coefficient in second-order factor model | | | | | | | | | | |
| Cognitive empathy |  |  |  |  |  | .1.00 | .60 |  |  |  |
| Affective empathy |  |  |  |  |  |  |  | .25 | .87 | .31 |
| Reliability | | | | | | | | | | |
| Omega | .92 | .90 | .72 | .77 | .25 | .92 | .90 | .72 | .77 | .25 |

ESEM-5 = five-factor ESEM; M4: H-ESEM-5 = Higher-order ESEM with five first-order factors; PT = Perspective taking; OS = Online simulation; EC = Emotion contagion; PRE = Peripheral responsivity; PRO = Proximal responsivity
