# Supplementary material for: Confirmatory factor analysis and exploratory structural equation modeling of the factor structure of the Questionnaire of Cognitive and Affective Empathy (QCAE)
Source: PLoS One. 2022 Feb 7;17(2):e0261914. doi: 10.1371/journal.pone.0261914 (PMC8820594; doi:10.1371/journal.pone.0261914)
Supplement: S3 File — (DOCX) [file pone.0261914.s003.docx]

**Confirmatory Factor Analysis and Exploratory Structural Equation Modelling of the Factor Structure of the Questionnaire of Cognitive and Affective Empathy (QCAE)**

The factor structure of the Questionnaire of Cognitive and Affective Empathy (QCAE) is challenged in the present study. It is argued that the proximal responsivity factor presents with low reliability. Therefore, an alternative four-factor structure is proposed. In addition, our findings showed that cognitive reappraisal was associated significantly and negatively with emotion contagion (EC), and significantly and positively with online simulation (OS) and peripheral responsivity (PER). Emotion suppression showed no association with any of the QCAE factors. Our findings have also reinforced positive associations for cognitive reappraisal with cognitive empathy whilst suggested also no association with affective empathy. Therefore, our findings show that while cognitive reappraisal (or strategies aimed at altering the way one is responding emotionally to the emotion-eliciting stimulus) is not associated with high levels of automatic mirroring of other’s feelings; It is associated with high levels of ability to put oneself in another person’s position by imagining what that person is feeling, and high ability to respond emotionally to the feelings of others in a film or a novel. In contrast, emotion suppression (or efforts to inhibit behaviours associated with the emotion being experienced) has no relation with empathy. These relations also indicate that cognitive empathy is adaptive, as has been proposed in the literature. In relation to shame and guilt, our findings showed that shame was associated significantly and negatively with perspective taking (PT), and significantly and positively with EC; as did guilt. These have implications for assessment, treatment as well as the theoretical conceptualization of Cognitive and Affective Empathy.
