# Supplementary figures and images for: Confirmatory factor analysis and exploratory structural equation modeling of the factor structure of the Questionnaire of Cognitive and Affective Empathy (QCAE)
Source: PLoS One. 2022 Feb 7;17(2):e0261914. doi: 10.1371/journal.pone.0261914 (PMC8820594; doi:10.1371/journal.pone.0261914)

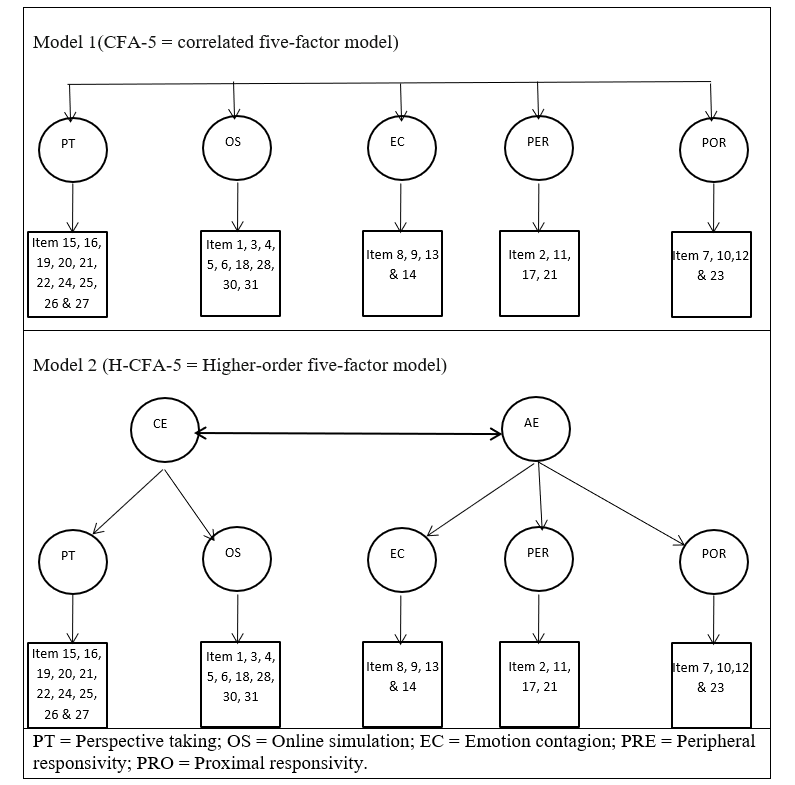

Supplement: S1 Fig — (TIF) [file pone.0261914.s004.tif]

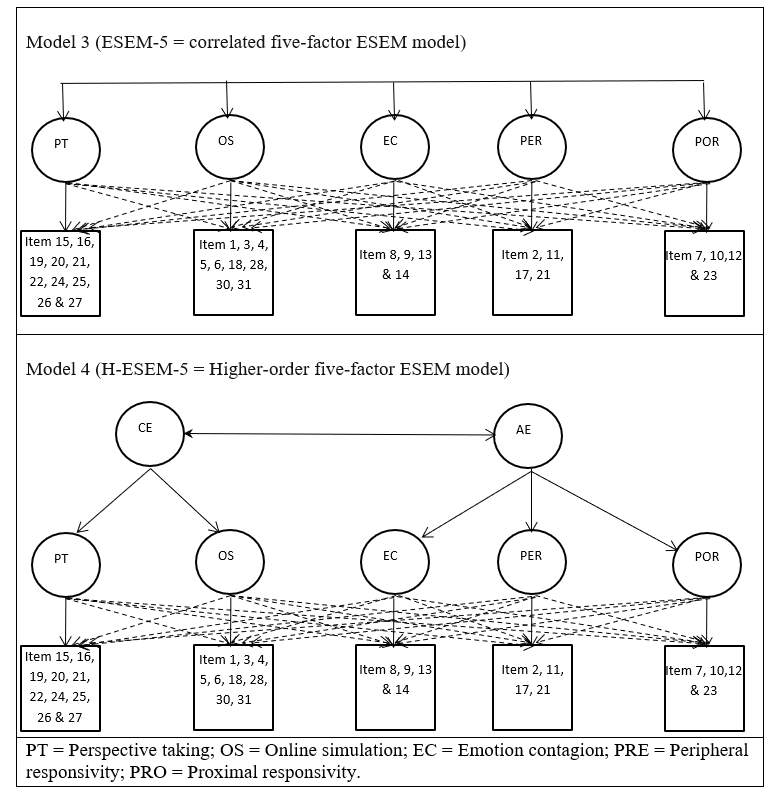

Supplement: S2 Fig — (TIF) [file pone.0261914.s005.tif]
