# Supplementary material for: Confirmatory factor analysis and exploratory structural equation modeling of the factor structure of the Questionnaire of Cognitive and Affective Empathy (QCAE)
Source: PLoS One. 2022 Feb 7;17(2):e0261914. doi: 10.1371/journal.pone.0261914 (PMC8820594; doi:10.1371/journal.pone.0261914)
Supplement: Attachment — Submitted filename: Response letter to editor and reviewer .docx [file pone.0261914.s006.docx]

09^th^ August 2021

Dr Meng-Cheng Wang
Academic Editor
PLOS ONE

Dear Dr. Meng-Cheng Wang,

Re: Confirmatory Factor Analysis and Exploratory Structural Equation Modeling of the Factor Structure of the Questionnaire of Cognitive and Affective Empathy (QCAE)
Ms Number: PONE-D-21-15654.

Thank you very much for arranging the review of the above mentioned paper that was submitted to PLOS ONE. We also like to thank the reviewers for the valuable comments. These have been extremely useful for the revision of the paper. In the new revised paper we have considered these comments, and we believe that we have responded to the revisions suggested. In relation to Review Comments to the Author (Question 5), our changes/revisions to them are detailed below.

**Reviewer #1:**

1 Introduction part, I can’t understand why you choose QCAE, it’s necessary? Why not choose IRI? It is used more popular. You should explain more.

**Response**: We have address this issue (p. 3, lines 48 o 56; yellow highlight)

2 Introduction part, ESEM with target rotation approach might good but why your choose this method? I don’t know. Because not be applied in any past literature?

**Response**: We have explained why we applied ESEM in the revised paper (p. 7, lines 150 to 155, tallow highlight).

3 Introduction part, if you want to test the external validity of the QCEA factors, why do you choose the Emotion Regulation Questionnaire and Test of Self-Conscious Affect-3? They are might correlated, but this evidence might not tough.

**Response**. We have now presented the reasons for using the ERQ and TOSCA-3 for testing the external validity of the QCEA (p. 9, lines 191 to 200; yellow highlight)

4 Method part, the author might supply more information about how do you decide the sample size. As far as I am concerned, the sample size was too small. In my opinion, you only examed that ESEM works better when you are a small sample size.

**Response**: The sample size issue is now covered (p.12, lines 264 to 271, yellow highlight)

5 There many spelling errors, such as QCEA, QCEAE.

**Response**: We have carefully checked and corrected the revised paper for all typo, grammar, and expression errors.

6 Whether QCAE must have five factors and two second-order factors? If not, why do we discuss the possible problems caused by the method? Why can't it be cultural differences or others?

**Response**: We have explained why the method used previously (CFA) may explain the findings related to past QCAE CFA findings (pp. 6 & 7, lines 131 to 139; yellow highlight).

**Reviewer #2:**

1. It seems that the sample size of the current study is a bit small. How this sample size can ensure adequate statistical power? The authors may consider using the power analysis to reveal whether the sample size of the current study can ensure the sufficient statistical power.

**Response:** We have conducted power analysis for this study (p.12, lines 264 to 271, yellow highlight)

2. Before conducting the reliability and validity analysis, I suggest that the authors add the item analysis of the QCAE.

**Response**. As suggested, we have discussed item analysis (i.e., item loadings) before conducting the reliability and validity analyses (p. 17, lines 365 to 378, green highlight)

3. There are several clerical errors in the Results section, please check carefully. For instance, the “Model 5” and “Model 6” (see Line 324) should be “Model 3” and “Model 4”; the “19” (see Line 328) should be “10”; the “21 stay emotionally detached” should be “29 stay emotionally detached” (see Line 332); “correlations in the six-factor model” should be “correlation in the five-factor model” in the Supplementary Table S2.

**Response**. All these have been corrected. Also, we have carefully checked and corrected the revised paper for all typo, grammar, and expression errors

4. According to the results, I recommend the author to take Table 3 instead of the Supplementary Table S2 in the manuscript.

**Response**. As suggested, Supplementary Table S2 has been moved to the main manuscript (now Tables 2 & 3).

5. It is suggested that the authors further explain and elaborate the revised four-factor model of the QCAE in the Discussion section.

**Response**. We have expanded and elaborated on the revised four-factor model of the QCAE in the Discussion section (pp. 23 & 24, lines 517 to 539; green highlight).

6. This study tested external validity of the QCAE using shame, guilt, cognitive reappraisal and emotion suppression, the authors might need to further add more the description for the external validity in the Discussion section.

**Response**. We have expanded and elaborated on our results testing external validity of the QCAE using shame, guilt, cognitive reappraisal and emotion suppression (p.25, lines 557 to 569; green highlight).

7. The format specification of references, more specifically, the month and date of each article in the Reference section need to be deleted.

**Response**. This has been complied with.

8. The Conclusion, Acknowledgments and Author Contribution section should be inserted just before the References.

**Response**. This has been complied with.

As you will notice we have addressed all the comments and suggestions made by the reviewers. We hope that the changes meet with your satisfaction and we look forward to hearing from you soon.

Thank you.

Yours sincerely,

Authors
