# Supplementary material for: Confirmatory factor analysis and exploratory structural equation modeling of the factor structure of the Questionnaire of Cognitive and Affective Empathy (QCAE)
Source: PLoS One. 2022 Feb 7;17(2):e0261914. doi: 10.1371/journal.pone.0261914 (PMC8820594; doi:10.1371/journal.pone.0261914)
Supplement: Peer review history

# Author comment: Author response to previous submission

**DOI:** 10.1371/journal.pone.0261914.r001

18 Sep 2021

Attachment Submitted filename: Response letter to editor and reviewer .docx Click here for additional data file.

# Editor report: Decision Letter 0

**DOI:** 10.1371/journal.pone.0261914.r002

**Authors:** Paola Gremigni

14 Dec 2021

Confirmatory Factor Analysis and Exploratory Structural Equation Modeling of the Factor Structure of the Questionnaire of Cognitive and Affective Empathy (QCAE)

PONE-D-21-29442

Dear Dr. Stavropoulos,

We’re pleased to inform you that your manuscript has been judged scientifically suitable for publication and will be formally accepted for publication once it meets all outstanding technical requirements.

Within one week, you’ll receive an e-mail detailing the required amendments. When these have been addressed, you’ll receive a formal acceptance letter and your manuscript will be scheduled for publication.

An invoice for payment will follow shortly after the formal acceptance. To ensure an efficient process, please log into Editorial Manager at http://www.editorialmanager.com/pone/, click the 'Update My Information' link at the top of the page, and double check that your user information is up-to-date. If you have any billing related questions, please contact our Author Billing department directly at authorbilling@plos.org.

If your institution or institutions have a press office, please notify them about your upcoming paper to help maximize its impact. If they’ll be preparing press materials, please inform our press team as soon as possible -- no later than 48 hours after receiving the formal acceptance. Your manuscript will remain under strict press embargo until 2 pm Eastern Time on the date of publication. For more information, please contact onepress@plos.org.

Kind regards,

Paola Gremigni, Ph.D.

Academic Editor

PLOS ONE

Journal Requirements:

1. Please note that in order to use the direct billing option the corresponding author must be affiliated with the chosen institute. Please respond by return e-mail so that we can amend your submission or remove this option. We can make any changes on your behalf.

Additional Editor Comments:

Dear Authors,

the manuscript improved thanks to your competent response to all the Reviewers' concerns. I particularly appreciated the effort you made in explaining better the statistic approach used.

Reviewers' comments:

# Editor report: Acceptance letter

**DOI:** 10.1371/journal.pone.0261914.r003

**Authors:** Paola Gremigni

28 Jan 2022

PONE-D-21-29442 

Confirmatory Factor Analysis and Exploratory Structural Equation Modeling of the Factor Structure of the Questionnaire of Cognitive and Affective Empathy (QCAE) 

Dear Dr. Stavropoulos:

I'm pleased to inform you that your manuscript has been deemed suitable for publication in PLOS ONE. Congratulations! Your manuscript is now with our production department. 

If your institution or institutions have a press office, please let them know about your upcoming paper now to help maximize its impact. If they'll be preparing press materials, please inform our press team within the next 48 hours. Your manuscript will remain under strict press embargo until 2 pm Eastern Time on the date of publication. For more information please contact onepress@plos.org.

If we can help with anything else, please email us at plosone@plos.org. 

Thank you for submitting your work to PLOS ONE and supporting open access. 

Kind regards, 

PLOS ONE Editorial Office Staff

on behalf of

Prof. Paola Gremigni 

Academic Editor

PLOS ONE
